# Unvarnished colonialism: America first is setting a dangerous precedent

**DOI:** 10.1371/journal.pmen.0000745

**Published:** 2026-10-09

**Authors:** Ariana Jaspal, Afifah Rahman-Shepherd, Dominique Aluquin, Nelson Aghogho Evaborhene, Shashika Bandara

**Affiliations:** 1 Independent Consultant, Toronto, Canada; 2 Saw Swee Hock School of Public Health, National University of Singapore, Singapore, Singapore; 3 Yong Loo Lin School of Medicine, National University of Singapore, Singapore, Singapore; 4 Centre for Interdisciplinary Study of Pandemic Signatures, Roskilde University, Roskilde, Denmark; 5 Department of Global Population Health, Jeffrey Cheah School of Medicine and Health Sciences, Monash University Malaysia, Subang Jaya, Malaysia; PLOS: Public Library of Science, UNITED KINGDOM OF GREAT BRITAIN AND NORTHERN IRELAND

Introduced in September 2025, the America First Global Health Strategy (AFGHS) came at the heels of cruel, abrupt US funding cuts that gutted local service delivery and research efforts, destabilized the global health financing architecture and undermined the principles of multilateralism [1,2]. The AFGHS has exploited the funding vacuum created by many high-income countries’ (HICs) withdrawal of development assistance for health (DAH): while espousing notions of leadership and partnership, the bilateral agreements target ‘recipient countries’ facing new fiscal pressures and aid uncertainty [3]. The “America First” approach reinforces emerging populist and isolationist global health strategies, and openly legitimizes a regression to colonial governance when wealthy colonizing nations made unjust demands from colonies in return for supporting basic human rights, including the right to health [4,5]. The AFGHS demands range from access to health data to high-value minerals, extending its influence far beyond health diplomacy to shape lives, care-seeking behavior, social inclusion, and overall wellbeing, and forcing us to face the imperial foundations that advance insidious and unethical agreements in global health.

Unpacking AFGHS sheds light on its colonial nature. Under the second pillar of AFGHS— to make the US ‘*stronger*’ — ‘recipient countries’ are expected to meet co-financing targets, absorb procurement risk, and share data without the option to negotiate terms, timelines, or any guarantee of reciprocity [3,6]. As made clear in the State Department’s remarks on the five-year agreement with Nigeria [7], funding is tied to compliance with US priorities, extending US oversight deep into countries’ health systems [8,9]. In the agreement with Nigeria, partly driven by domestic electoral politics in the US, the Strategy shifts towards faith-based health service delivery, using a colonial tactic that fuses religious dominance with governance structures and administration [7]. While faith and spirituality can play important roles in health and wellbeing, the prioritization of faith-based organizations within AFGHS that align with US foreign policy and the subsequent domestic shifts in religious exclusion could do more harm than good and aggravate existing social tensions [10,11]. This shift reshapes both healthcare delivery and the scope of permissible care, severely restricting rights-based and non-faith based (or secular) health services and workers. The impacts of this change are particularly harmful for LGBTQ+ individuals and women seeking life-saving reproductive care, building on the US’ attacks on global reproductive rights [10,12]. It will also significantly constrain sexual and reproductive health care, with moralized framings leading to greater stigma and self-exclusion among people accessing these services. These harms leave vulnerable communities without discrete and affirming services, heightening the potential risk of anxiety and depression and demonstrating the structural mental health and wellbeing costs embedded in the AFGHS.

Arguably the most explicitly colonial agenda is conditioning financing for health on access to strategic resources. In Zambia, the US threatened to withhold financing unless granted preferential access to critical renewable energy and technology minerals such as copper, cobalt, and lithium [6,13]. But AFGHS largely centers around the extraction of an ‘invisible’ resource: health data. Under the third pillar of AFGHS— to make the US *‘more prosperous’* — ‘recipient countries’ are expected to supply surveillance data and biological specimens and procure US health products and innovations. Health data is a national asset with implications for countries’ health sovereignty, but the extraction of this data makes it unlikely that the countries who provide it can “hold intellectual property, shape research agendas, or reliably access the resulting products” [3]. Under AFGHS conditions, ‘recipient countries’ feed US intelligence while their health systems become structurally oriented around US supply chains, undermining the aim of self-reliance AFGHS claims to pursue. The routine capture of health data also has grave ethical and wellbeing implications, reducing patient’s confidence and awareness of where, how, and by whom their data will be stored, processed, and used. This is particularly harmful for individuals accessing services for stigmatized concerns, such as mental, sexual, and reproductive health, where perceived confidentiality is crucial to build the trust needed to encourage people to safely seek care without fear of social judgment or discrimination [14]. The extractive nature of AFGHS is fundamentally incompatible with the development of long-term care systems that are required to address physical and mental health and wellbeing [15]. When care systems rely on transactional arrangements, they create dependencies that leave communities vulnerable to any shifts in the demands or conditions of that arrangement.

The AFGHS sets a dangerous precedent in an already fragile global health governance system, that health assistance no longer needs to be presented as a global public good, but as a strategic asset that can be deployed and leveraged in pursuit of national advantage regardless of cost [8]. The AFGHS effectively lowers the bar for what can be considered ‘global health’, signaling an unabashed return to structural extractivism that is destructive to the future of our field, which relies on stable, locally-driven, and context-specific systems, rather than arrangements that shift with geopolitical interests. Our usual rhetoric of solidarity and ‘soft’ diplomacy to manage powerful actors will not suffice. If AFGHS signals a shift toward more openly transactional and extractive arrangements in global health, global health reforms must include counter arrangements that consider negotiation of power within global governance as a factor. This negotiation must include self-sufficiency of countries by strengthening their own contribution to national health budgets, regional manufacturing efforts, and health infrastructure. There are lessons from the anti-colonial, pan-regional movements that spread across the Global South in the 20th century – the legacy of which is evident today in the African Union and the Association of Southeast Asian Nations – and Kenya’s high court ruling against AFGHS and Zambia’s rejection of its extractive conditions, which demonstrate how countries are increasingly contesting the terms of cooperation, asserting greater control over their priorities, resources, and data [16]. Reform proposals must deliver health sovereignty and subsidiarity-based models of governance, to enable countries to effectively mobilise domestic resources for health and to develop regional institutions as a more reliable and sustainable layer of support. Achieving health sovereignty and negating the colonial models of health governance is ultimately about ensuring every human, regardless of where they live, have the freedom and the capacity to access the health care they need. Only then can lives and livelihoods be protected from becoming geopolitical bargaining chips.
